# Predicting adult mental health from non-suicidal self-injury in adolescence: a prospective study spanning 2007–2023

**DOI:** 10.1007/s00787-025-02785-8

**Published:** 2025-06-11

**Authors:** Benjamin Claréus, Margit Wångby-Lundh, Lars-Gunnar Lundh, Gustaf Rådman, Jonas Bjärehed, Daiva Daukantaitė

**Affiliations:** 1 Department of Psychology, Kristianstad University, Kristianstad, 291 88 Sweden; 2 Department of Psychology, Lund University, Lund, 221 00 Sweden; 3 Department of Clinical Sciences Lund, Lund University, Lund, 221 84 Sweden

**Keywords:** Longitudinal, Prospective, Non-suicidal self-injury, Adulthood, Adolescence

## Abstract

This study examined associations between adolescent non-suicidal self-injury (NSSI) and adult mental health within a cohort of school students who answered questionnaires in 2007 (T1: *N* = 992, *M*_age_ = 13.7, 50.1% girls) and 2008 (T2: *N* = 987, *M*_age_ = 14.9, 50.9% girls), with follow-ups in 2017 (T3: *N* = 557, *M*_age_ = 25.3, 58.7% women) and 2023 (T4: *N* = 386, *M*_age_ = 29.9, 63.0% women). Attrition was accounted for with sample weighting and mixed modelling, and robust estimates balance for residual non-normality. Significant declines in any NSSI (≥ 1 instance) and repetitive NSSI (≥ 5 instances) were observed between T2–T3 (any NSSI: 43–19%; repetitive NSSI: 21–10%; *p* <.001), but not T3–T4 (any NSSI: 19% to around 14%; repetitive NSSI: 10–5%; *p* ≥.078). Compared to reporting *no NSSI* in adolescence, *stable repetitive NSSI* (≥ 5 instances at T1 and T2), *unstable repetitive NSSI* (≥ 5 instances at T1 or T2), and *infrequent NSSI* (1–4 instances at T1 and/or T2) were significantly associated with any NSSI engagement (*OR* = 2.63–6.67) and elevated depression, anxiety, and stress levels at T3/T4 (*β*_robust_ = 0.14–0.39), while controlling for gender and adolescent psychological difficulties. For *stable repetitive NSSI*, this applied to emotion dysregulation as well (*β*_robust_ = 0.44). Additionally, *stable/unstable repetitive NSSI* was significantly associated with lower well-being when not controlling for gender and psychological difficulties (*β*_robust_ =–0.32−-0.41). The findings indicate that adolescent NSSI, especially when repetitive and stable, predicts poorer health across the first decade of adulthood.

**Supplementary Information:**

The online version contains supplementary material available at 10.1007/s00787-025-02785-8.

Non-suicidal self-injury (NSSI) refers to intentional behaviours that cause direct damage to one’s body (e.g., cutting or hitting oneself) without suicidal intent (e.g [1]). Adolescents who self-injure often experience elevated levels of psychological distress, especially among those where engagement is repetitive (i.e., ≥ 5–6 times; [2]). Our earlier findings [3] suggested that infrequent and repetitive engagement in NSSI during adolescence (mean age = 13.7–14.9 years) each had unique prospective associations with mental health variables 10 years later (mean age = 25.3 years). The current study builds on our previous work by including a fourth wave of data collection from the same cohort (mean age = 29.85 years). The purpose was to estimate the prevalence of NSSI in adulthood proper, and to further investigate how different frequency patterns of adolescent NSSI (i.e., infrequent, unstable repetitive, and stable repetitive) are related to mental health in adulthood proper.

In addition to our study (“Självkänsla Och Livssituation”; SOL), there are to our knowledge six other longitudinal studies of adolescent NSSI that have conducted follow-ups in adulthood (i.e [4], plus ALPAC, e.g [5, 6], Y@H/SHoT2018 [7], VAHCS, e.g [8–10]. V-HYS [11], and z-proso [12]). These studies, which all relied on a single-item measure of adolescent NSSI, show that self-reported NSSI in adolescence is associated with increased risk for several negative mental health and behavioural outcomes in adulthood. For example, adolescent NSSI was found to be associated with a diagnosis of depression and anxiety three [5], six [7], and twenty years later [8], and with increased risk for lower well-being in terms of environmental mastery and self-acceptance at around 25 years of age [11]. Additionally, reporting NSSI in adolescence has been associated with high-risk alcohol consumption and illicit substance use in adulthood [8, 10], and with NSSI engagement around 21–25 years [4, 5, 7].

In line with these findings, results from the SOL study [3] have suggested that repetitive NSSI in adolescence (i.e., reporting ≥ 5 instances of NSSI in 2007 and/or 2008) is associated with increased risk for elevated stress and anxiety levels 10 years later (in 2017). However, only stable repetitive NSSI (i.e., repetitive NSSI in *both* 2007 and 2008) was associated with increased risk for NSSI engagement and emotion dysregulation. By utilizing a behavioural checklist measuring frequencies of different self-injurious behaviours, we could also account for individuals who self-injured less frequently, and who are often misclassified as not currently self-injuring by single-item NSSI measures [13]. Unlike repetitive adolescent NSSI, infrequent adolescent NSSI (i.e., reporting 1–4 instances in 2007 and/or 2008) was not consistently predictive of ill-being after controlling for other relevant variables. However, like repetitive NSSI, infrequent NSSI was associated with emotion dysregulation as well as with elevated levels of depression, anxiety, and stress in a model that did not include other predictors. Further research is needed to examine whether these associations between adolescent NSSI and future mental health problems persist into later adulthood as well, especially since only one previous study of adolescent NSSI has conducted a follow-up in adulthood proper (i.e., [8]).

In the present study, our aims were twofold. First, we wanted to estimate the prevalence rates of any NSSI and of repetitive NSSI in adulthood proper (30 years old) and compare these with estimates from adolescence (13–15 years old) and young adulthood (25 years old). In line with Moran et al. [9], we expected that the declining prevalence rates previously observed from adolescence to young adulthood (c.f., [3]), would weaken, resulting in similar estimates at age 30 as at age 25.

Our second aim was to explore if different frequency patterns of adolescent NSSI could predict NSSI and aspects of mental health across the first decade of adulthood. We hypothesized that infrequent NSSI and unstable/stable repetitive NSSI during adolescence would be associated with an increased risk of NSSI in adulthood (i.e., ≥ 1 instances) (c.f [3–5]). We also tested whether the adolescent NSSI patterns could predict level of NSSI engagement among the participants who reported NSSI in adulthood. Furthermore, we hypothesized that adolescent NSSI and particularly repetitive engagement would be associated with higher levels of depression, anxiety and stress along with lower levels of flourishing and life satisfaction and greater emotion dysregulation (c.f [3, 11]). We also intended to explore differential change in these associations during adulthood. For example, this included examining whether stable repetitive NSSI during adolescence had a stronger or weaker association with aspects of mental health when the sample was 25 as compared to 30 years old. As no previous studies have investigated this, we did not formulate hypotheses regarding differential change.

## Methods

### Participants

Data were collected in spring 2007 (T1), spring 2008 (T2), autumn 2017 (T3), and spring 2023 (T4). At T1, eligible participants were students in Grade 7 or Grade 8 in all regular schools within a southern Swedish municipality of about 40,000 residents. At T2, data was collected from Grade 8 and Grade 9 students in the same schools.

The study relies on self-reported gender so that participants could select if they identified as a *girl/woman* (T1–T4), *boy/man* (T1–T4), or *other/does not want to disclose* (T3–T4). In the current study, participant gender has been adjusted retrospectively to align with the most recent observation.^1^ In total, 992 students participated at T1 (response rate: 93.2%, mean [*SD*] age = 13.73 [0.68] years; 50.1% girls, 49.7% boys, and 0.2% other), and 987 students participated at T2 (response rate: 89.9%; mean age [*SD*] = 14.87 [0.69] years; 50.9% girls, 48.9% boys, 0.2% other). Data collection at T3 and T4 addressed all participants eligible at the previous two data collection points (*N* = 1109). The sample was comprised of 557 individuals at T3 (response rate: 50.2%; mean age [*SD*] = 25.33 [0.68] years; 58.7% women, 41.1% men, 0.2% other), and 386 individuals at T4 (response rate: 34.8%; mean age [*SD*] = 29.85 [0.75] years; 63.0% women, 36.5% men; 0.5% other). Detailed demographic data from T3 and T4 is available in Table A1 of the Supplement.

**Fig. 1:**
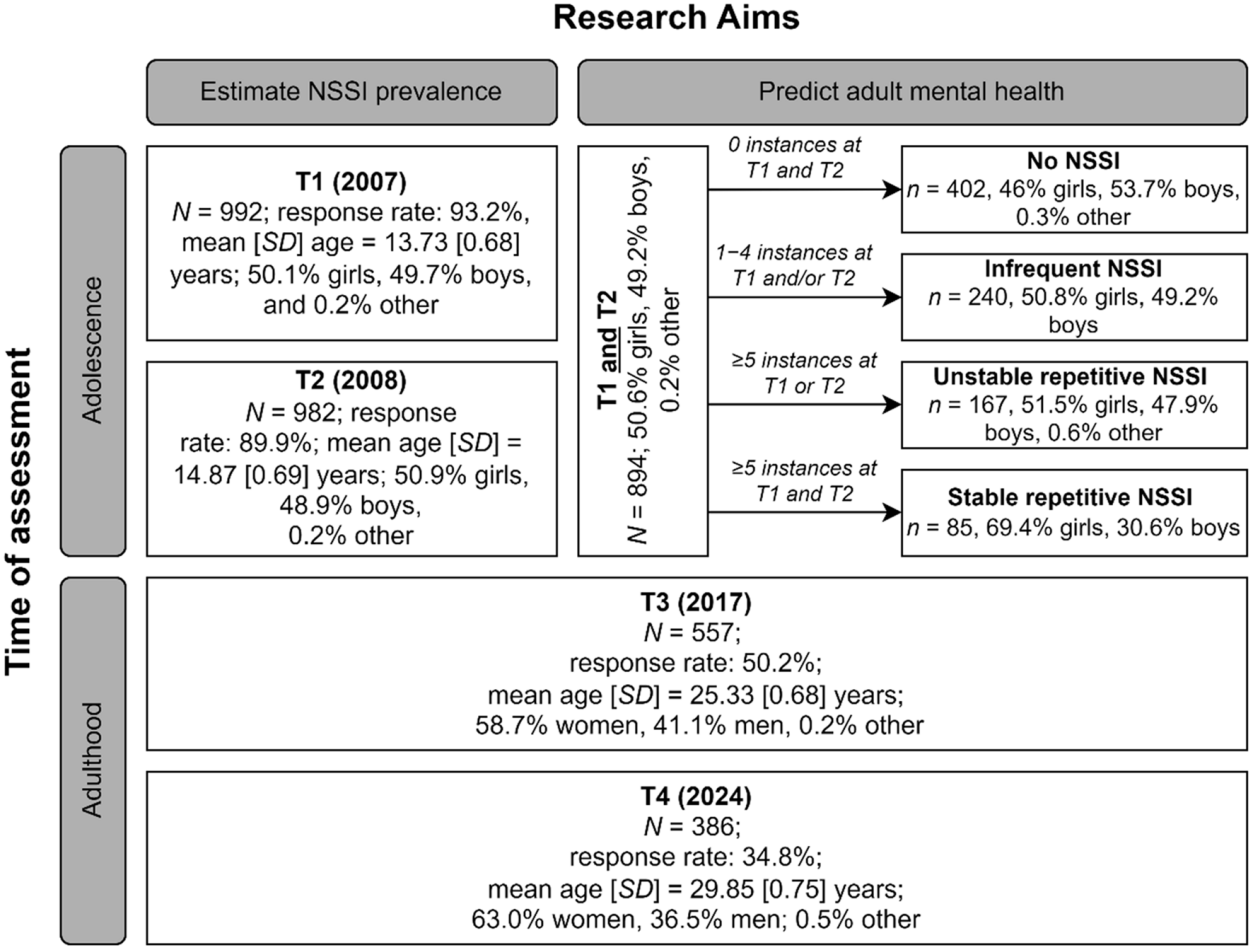
Sample overview. NSSI = Non-suicidal self-injury

In this study, NSSI prevalence was estimated utilizing data from all participants as described above. As both T1 and T2 data were necessary for constructing the adolescent NSSI frequency patterns, outcome analysis only included those 894 participants who had responded at T1 plus T2 and had valid NSSI data at both these time points. An overview is provided in Fig. 1.

## Measures

### NSSI in adolescence and adulthood (T1–T4)

NSSI was measured with the revised Deliberate Self-Harm Inventory (DSHI-9r; [14]). Using a scale from 0 (*never*) to 6 (*more than five times*), respondents are asked to rate how often they have intentionally engaged in any of nine self-injurious behaviours, including: (1) cutting wrists, arms or other body areas; (2) surface cutting, causing a wound with some bleeding; (3) punching/banging one’s head; (4) carving words, pictures, etc. into skin; (5) severe scratching, causing bleeding; (6) burning with cigarette, lighter or match; (7) sticking sharp objects into skin; (8) biting oneself, so that skin is broken; and (9) preventing wounds from healing. The relevant time frame was within the past 6 months at T1/T2, and within 12 months at T3/T4. A total score (range 0–54) was computed by summing all items, and the Cronbach’s alphas were 0.90 (T1), 0.90 (T2), 0.81 (T3), and 0.77 (T4).

In a previous publication [3], total scores on the DSHI-9r at T1 and T2 were used to establish the different frequency patterns of adolescent NSSI that are used here. These include *no NSSI* (0 instances at T1 and T2), *infrequent NSSI* (1–4 instances at T1 and/or T2), *unstable repetitive NSSI* (≥ 5 instances at T1 or T2), and *stable repetitive NSSI* (≥ 5 instances at T1 and T2). Raw data on adulthood NSSI engagement for individuals with different frequency patterns is reported in Table A2 in the Supplement. A separate question asking whether participants had attempted suicide as they injured themselves was included at T3 and T4.

### Psychological difficulties in adolescence (T1–T2)

The Strengths and Difficulties Questionnaire–self-report version (SDQ-s; [15]) was used to measure psychological difficulties. The SDQ-s entails 20 questions related to hyperactivity-inattention, emotional symptoms, conduct problems, and peer problems as experienced within the past 6 months, which participants rate on a 3-point Likert scale (0 = *not true*; 2 = *certainly true*). The Cronbach’s alphas were 0.76 (T1) and 0.75 (T2).

### Mental health indicators in adulthood (T3–T4)

*Depression*, *anxiety*, and *stress* as experienced within the last month were measured using the 21-item version of the Depression, Anxiety, and Stress Scale [16], where participants rate 7 items pertaining to each construct on a scale from 0 (*never*) to 3 (*almost always*). For each subscale, the Cronbach’s alphas at T3 and T4 were as follows: depression (*α*_T3_ = 0.90; *α*_T4_ = 0.91), anxiety (*α*_T3_ = 0.79; *α*_T4_ = 0.77), and stress (*α*_T3_ = 0.87; *α*_T4_ = 0.84).

*Life satisfaction* and *flourishing* were measured with the 5-item Satisfaction with Life Scale (SWLS; [17]) and the 8-item Flourishing Scale (FS; [18]), respectively. Flourishing describes a state of emotional, psychological, and social well-being, and in the FS, it is operationalized through positive emotion, engagement, meaningful relationships, and optimism [18]. Both scales use a 7-point Likert scale from 1 (*strongly disagree*) to 7 (*strongly agree*) and had high Cronbach alphas (SWLS: *α*_T3_ = 0.92, *α*_T4_ = 0.93; FS: *α*_T3_ = 0.88, *α*_T4_ = 0.93).

*Emotion dysregulation* was measured using the 16-item Brief Difficulties in Emotion Regulation Scale [19]. Participants answer on a scale from 1 (*almost never/0–10% of the time*) to 5 (*almost always/91–100% of the time*). The Cronbach’s alpha was 0.95 at both T3 and T4.

### Procedure

Data collection at T1 and T2 was conducted by research assistants from Lund University and took place in schools during a separate lecture hour. Prior to data collection, information about the study and its purposes was sent to parents/guardians and handed out to the students. This information, which emphasised the voluntary nature of participation and the right to withdraw, was also presented verbally before the questionnaires were distributed. Students consented to participation and could terminate the survey or hand in a blank questionnaire. Parents/guardians of students younger than 15 years needed to contact the project leader or school staff to withdraw their child from the study. All questionnaires were filled out by paper-and-pencil, and numeric codes were used to match the data at the different time points according to a pseudonymized procedure.

To conduct follow-ups at T3 and T4, each participant’s unique personal identity number was sent to the Swedish state’s personal address register, which returned information about their current address. Letters detailing the purpose of the study and the context of why they were contacted were sent to this address, with either personalized login credentials to an encrypted online survey or a paper-and-pencil questionnaire that could be returned by mail. Participation was reimbursed with a 150–200 SEK gift. Participant’s personal details were not written anywhere else than on the envelope in which they received information about the study; instead, numeric codes were used to match the data longitudinally. Participants could withdraw consent by choosing to not answer any questions, or they could contact the researchers directly to be removed from the participation pool.

### Statistical analysis

Due to high attrition rates between adolescence to adulthood, we tested for differences between responders and non-responders at both T3 and T4 [20, 21]. Any significant differences in the available data were small (Cohen’s *d*/Cramer’s *V* = 0.07–0.28). Women were more likely than men to respond at T3 (58.4% vs. 41.9%; χ^2^ = 30.19, *p* <.001) and T4 (43.4% vs. 25.8%; χ^2^ = 37.25, *p* <.001). Adolescent NSSI frequency pattern was associated with attrition at T3 (χ^2^ = 11.86, *p* <.008) but not at T4 (χ^2^ = 2.12, *p* =.549). Those reporting *stable repetitive NSSI* were overrepresented (62.4% answered) and those reporting *unstable repetitive NSSI* were underrepresented (42.5% answered) among the responders at T3. No other study variables assessed at a previous time point were significantly associated with attrition at T3 or T4 (*p* >.220).

To account for attrition, prevalence estimates at T3–T4 were made more representative of the cohort by increasing the weight of responders who were similar to non-responders in terms of gender, adolescent psychological difficulties, and adolescent NSSI frequency pattern. Furthermore, the mixed effects models (described below) were fitted on all available data (i.e., included both responders and non-responders at T3–T4) with participant ID included as a random intercept, after occasional missing scale items had been imputed with expectation-maximization–or, in the case of DSHI-9r, replaced with 0 if participants had 6 or more valid answers (in accordance with previous publications; e.g., [3, 14]).

Zero-inflated Poisson mixed models (using a hybrid expectation-maximization/quasi-Newton optimization approach) were used to test the associations between adolescent NSSI frequency patterns and NSSI in adulthood. This model is designed for analysing count data with an excess of zero values [22], which is the case of DSHI-9r. It combines two components: a zero-inflation component that accounts for structural zeros (the likelihood of an event *not* occurring; i.e., the likelihood of *not* self-injuring during the past year in adulthood), and a Poisson component for modelling non-zero scores (the frequency of an event after excluding zero scores; i.e., level of NSSI engagement among participants who reported ≥ 1 instances in adulthood). For the other outcomes, restricted maximum-likelihood linear mixed models with robust estimates (calculated subsequent of assigning a lower weight to high-residual observations; [23]) were utilized. Three models were evaluated for each outcome. The first model included time of assessment (0 = *2017*/*T3*, 1 = *2023*/*T4*) and adolescent NSSI frequency pattern (with *no NSSI* as the reference group; c.f [3])., as fixed effects, and the second added participant gender (0 = *boy/man*, 1 = *girl/woman*; *other* was set as missing due to small sample size) and standardized mean score in psychological difficulties across T1–T2. These covariates were added due to observed differences in NSSI prevalence between girls/women and boys/men, and previous longitudinal studies similarly have included internalizing/externalizing problems as covariates [5, 8, 11]. The third model included an interaction term between time of assessment and adolescent NSSI frequency pattern, and *p*-values were Bonferroni corrected as no hypotheses had been formulated ad hoc.

## Results

### Changes in NSSI prevalence over time

As shown in Table 1, both the weighted and unweighted prevalence rates of any NSSI (≥ 1 instance) and repetitive NSSI (≥ 5 instances) appear to decline between T2 and T3 (43–19% for any NSSI; 21–10% for repetitive NSSI), as well as between T3 and T4 (19% to 14–15% for any NSSI; 10–5% for repetitive NSSI). However, confidence intervals (*CI*s) for the T3 prevalence rates overlapped with the T4 intervals; accordingly, McNemar tests suggested that while the decline was statistically significant between T2 to T3 (*p* <.001), this was not the case between T3 to T4 (*p*_any NSSI_ = 0.175; *p*_repetitive NSSI_ = 0.078). Girls/women were significantly more likely than boys/men to self-injure at T1–T3 (*χ*^*2*^ = 3.91–14.81, *p* =.001–.048) but not at T4 (*χ*^*2*^ = 1.74, *p* =.187). Regarding repetitive NSSI, differences between girls/women and boys/men were only significant at T2 (*χ*^*2*^ = 14.20, *p* <.001; repetitive NSSI was more prevalent among girls/women) but not at any other time point (*χ*^*2*^ = 0.43–2.96, *p* =.085–.512). Three participants at T3 and one participant at T4 reported that they had attempted suicide when they injured themselves.

### Adolescent NSSI frequency patterns and NSSI in adulthood

As presented in Table 2, when predicting the likelihood of *not* self-injuring at all (i.e., the zero-inflation part), NSSI in adolescence was significantly associated with any NSSI engagement in adulthood, even after controlling for gender and adolescent psychological difficulties. In this adjusted model, *infrequent NSSI* was associated with lower odds of not engaging in NSSI at all in adulthood (*OR* [95% *CI*] = 0.25 [0.10, 0.57]; equal to *OR* = 1/0.25 = 4.00 for engaging in NSSI), as were *unstable repetitive NSSI (OR* = 0.38 [0.18, 0.80]; *OR* = 2.63 for NSSI engagement) and *stable repetitive NSSI (OR* = 0.15 [0.07, 0.36]; *OR* = 6.67 for NSSI engagement). There was no significant interaction effect (Supplement, Table A3).

**Fig. 2:**
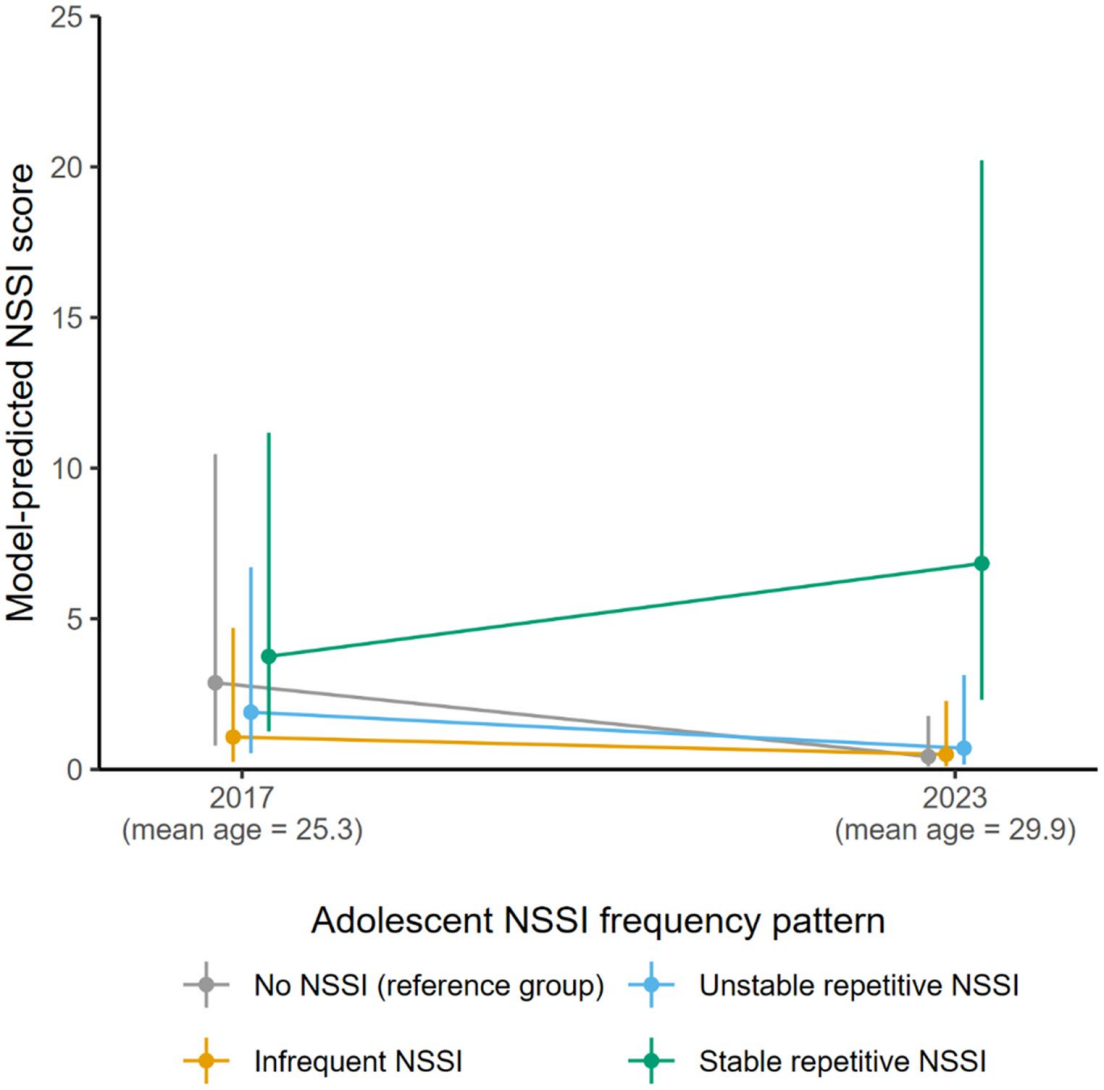
Model-predicted non-suicidal self-injury (NSSI) scores and asymptotic confidence intervals in young adulthood and adulthood proper among individuals currently self-injuring, over four adolescent NSSI frequency patterns (i.e., time-of-assessment×NSSI frequency pattern). Predictions are on the response scale and based on the count-part of a zero-inflated Poisson regression model while adjusting for gender and adolescent psychological difficulties (see Supplement, Table A3)

When focusing solely on participants who did report NSSI in adulthood (i.e., in the Poisson regression component), neither NSSI frequency pattern had a main effect on level of engagement once covariates were included. Nevertheless, statistically significant interaction effects with time of assessment were observed for all frequency patterns. Figure 2 suggests that the level of engagement among participants who self-injured was comparable between 2017 and 2023 for the *stable repetitive NSSI* group. In contrast, for participants who self-injured and belonged to the *infrequent NSSI* and *unstable repetitive NSSI* groups, the average level of engagement decreased between the ages of 25 and 30.

### Adolescent NSSI frequency patterns and mental health indicators in adulthood

Table 3 suggests that even when gender and psychological difficulties were controlled for, each of the three adolescent NSSI frequency patterns were significantly associated with higher scores in depression (adjusted: *β*_robust_ = 0.18–0.29; non-adjusted: *β*_robust_ = 0.25–0.46), anxiety (adjusted: *β*_robust_ = 0.17–0.39; non-adjusted: *β*_robust_ = 0.23–0.61), and stress (adjusted: *β*_robust_ = 0.14–0.30,: non-adjusted: *β*_robust_ = 0.24–0.65). On the other hand, negative associations to life satisfaction and flourishing for the *unstable/stable repetitive NSSI* patterns (non-adjusted: *β*_robust_ =–0.31−-0.42, *p* ≤.005) were weakened and nonsignificant in the adjusted model. This also applied to associations with emotion dysregulation for the *infrequent NSSI* (non-adjusted: *β*_robust_ = 0.19, *p* =.017; adjusted: *p* =.141) and *unstable repetitive NSSI* patterns (non-adjusted: *β*_robust_ = 0.39, *p* <.001; adjusted: *β*_robust_ = 0.18, *p* =.061). However, *stable repetitive NSSI* retained a significant association with emotion dysregulation (adjusted: *β*_robust_ = 0.44; non-adjusted: *β*_robust_ = 0.82). No significant changes over assessments were suggested at the sample level (*p* ≥.561), and exploratory follow-up tests did not support any significant interactions between time of assessment and adolescent NSSI frequency pattern (Supplement, Table A4).

**Table 1:** Prevalence (%) and mean for NSSI at the four measurement points

Time of assessment	Unweighted estimates– *n*/*N* (%) or M (SD)			Weighted estimates– % [95% CI] or M [95% CI]		
	Total	Girls/women	Boys/men	Total	Girls/women	Boys/men
Any NSSI (≥ 1 instances)						
2007 (T1)	408/982 (41.5%)	221/493 (44.8%)	187/487 (38.4%)			
2008 (T2)	420/979 (42.9%)	243/497 (48.9%)	176/482 (36.5%)			
2017 (T3)	104/556 (18.7%)	72/327 (22%)	32/228 (14%)	18.8% [15.5, 23]	21% [16.8, 26]	16% [11.8, 23]
2023 (T4)	58/386 (15%)	41/243 (16.9%)	16/141 (11.3%)	14.1% [10.8, 18]	16.8% [12.5, 22]	10.9% [6.5, 18]
Repetitive NSSI (≥ 5 instances)						
2007 (T1)	181/982 (18.4%)	102/493 (20.7%)	79/487 (16.2%)			
2008 (T2)	205/979 (20.9%)	128/497 (25.8%)	76/482 (15.8%)			
2017 (T3)	58/556 (10.4%)	37/327 (11.3%)	21/228 (9.2%)	10.5% [8, 14]	10.5% [7.6, 14]	10.6% [6.8, 16]
2023 (T4)	21/386 (5.4%)	17/243 (7%)	4/141 (2.8%)	5.1% [3.2, 8]	6.8% [4.2, 11]	3.4% [1.2, 10]
NSSI, summed score						
2007 (T1)	3.47 (8.07)	4.23 (9.18)	2.72 (6.7)			
2008 (T2)	3.78 (8.85)	4.64 (9.3)	2.9 (8.29)			
2017 (T3)	1.45 (4.82)	1.69 (5.09)	1.11 (4.39)	1.50 [0.96, 2.05]	1.53 [1.04, 2.02]	1.48 [0.48, 2.47]
2023 (T4)	0.78 (3.14)	1.05 (3.83)	0.30 (1.13)	0.68 [0.42, 0.94]	1.02 [0.56, 1.48]	0.31 [0.10, 0.53]

Note. NSSI was assessed within the past 6 months (T1–T2) or past 12 months (T3–T4). The weighted estimates increase the weight of responders who were similar to the non-responders on gender, psychological difficulties, and NSSI at T1/T2. NSSI = Non-Suicidal Self-Injury

**Table 2:** Predicting NSSI in adulthood (2017–2023) from NSSI in adolescence (2007–2008) with zero-inflated Poisson mixed modelling

Predictors	Zero-inflated component			Poisson regression component	
	α (SE)	OR [95% CI]	*p*	b (SE)	*p*
**Model 1:**					
Assessment (0 = *2017*, 1 = *2023*)	0.03 (0.25)	1.03 [0.62, 1.69]	0.911	− 0.43 (0.32)	0.188
Reference: No NSSI					
Infrequent NSSI	-1.52 (0.48)	0.22 [0.08, 0.57]	0.002	− 0.72 (0.54)	0.187
Unstable repetitive NSSI	-1.12 (0.37)	0.33 [0.16, 0.68]	0.003	− 0.25 (0.46)	0.584
Stable repetitive NSSI	-2.27 (0.36)	0.10 [0.05, 0.21]	< 0.001	0.92 (0.45)	0.039
**Model 2:**					
Assessment (0 = *2017*, 1 = *2023*)	0.07 (0.25)	1.07 [0.65, 1.76]	0.797	− 0.43 (0.33)	0.190
Reference: No NSSI					
Infrequent NSSI	-1.41 (0.43)	0.25 [0.10, 0.57]	0.001	− 0.68 (0.52)	0.191
Unstable repetitive NSSI	− 0.98 (0.39)	0.38 [0.18, 0.80]	0.011	− 0.30 (0.49)	0.543
Stable repetitive NSSI	-1.88 (0.43)	0.15 [0.07, 0.36]	< 0.001	0.84 (0.48)	0.081
Psychological difficulties in adolescence	− 0.25 (0.13)	0.78 [0.60, 1.01]	0.056	0.01 (0.15)	0.922
Gender (0 = *Boy/man*, 1 = *Girl/Woman*)	− 0.27 (0.30)	0.76 [0.42, 1.38]	0.368	0.11 (0.35)	0.762

Note. The zero-part predicts likelihood of *not* self-injuring. NSSI frequency patterns in adolescence were mutually exclusive dummy coded variables. Psychological difficulties were operationalized as the mean score over 2007–2008 for the Strength and Difficulties Questionnaire–self-report version. NSSI = Non-Suicidal Self-Injury

**Table 3:** Predicting mental health and emotion regulation in adulthood (2017–2023) from NSSI in adolescence (2007–2008) with mixed linear modelling

Predictors	Depression			Anxiety			Stress		
	β (SE)	β_robust_	*p*	β (SE)	β_robust_	*p*	β (SE)	β_robust_	*p*
**Model 1:**									
Assessment (0 = *2017*, 1 = *2023*)	− 0.02 (0.06)	0.00	0.795	0.00 (0.06)	− 0.01	0.993	− 0.02 (0.06)	− 0.02	0.680
Reference: No NSSI									
Infrequent NSSI	0.30 (0.09)	0.25	0.001	0.29 (0.09)	0.23	0.001	0.28 (0.09)	0.24	0.002
Unstable repetitive NSSI	0.51 (0.11)	0.43	< 0.001	0.47 (0.11)	0.36	< 0.001	0.51 (0.11)	0.52	< 0.001
Stable repetitive NSSI	0.58 (0.13)	0.46	< 0.001	0.72 (0.13)	0.61	< 0.001	0.68 (0.13)	0.65	< 0.001
**Model 2**:									
Assessment (0 = *2017*, 1 = *2023*)	− 0.03 (0.06)	− 0.01	0.597	− 0.02 (0.06)	− 0.03	0.732	− 0.05 (0.06)	− 0.05	0.392
Reference: No NSSI									
Infrequent NSSI	0.24 (0.09)	0.18	0.006	0.21 (0.09)	0.17	0.020	0.19 (0.09)	0.14	0.033
Unstable repetitive NSSI	0.35 (0.11)	0.29	0.001	0.30 (0.11)	0.25	0.007	0.32 (0.11)	0.30	0.004
Stable repetitive NSSI	0.29 (0.14)	0.22	0.034	0.39 (0.14)	0.39	0.005	0.28 (0.14)	0.20	0.048
Psychological difficulties in adolescence	0.17 (0.04)	0.15	< 0.001	0.18 (0.04)	0.13	< 0.001	0.19 (0.04)	0.21	< 0.001
Gender (0 = *Boy/man*, 1 = *Girl/Woman*)	0.11 (0.07)	0.11	0.142	0.15 (0.07)	0.10	0.039	0.34 (0.07)	0.32	< 0.001
	Life satisfaction			Flourishing			Emotion dysregulation		
	*β* (*SE*)	*β*_robust_	*p*	*β* (*SE*)	*β*_robust_	*p*	*β* (*SE*)	*β*_robust_	*p*
**Model 1:**									
Assessment (0 = *2017*, 1 = *2023*)	− 0.02 (0.05)	− 0.02	0.740	0.03 (0.05)	0.05	0.561	− 0.03 (0.05)	− 0.05	0.567
Reference: No NSSI									
Infrequent NSSI	− 0.12 (0.09)	− 0.10	0.220	− 0.15 (0.09)	− 0.15	0.116	0.22 (0.09)	0.19	0.017
Unstable repetitive NSSI	− 0.35 (0.12)	− 0.32	0.003	− 0.33 (0.12)	− 0.31	0.005	0.40 (0.11)	0.39	< 0.001
Stable repetitive NSSI	− 0.40 (0.14)	− 0.41	0.003	− 0.47 (0.14)	− 0.42	0.001	0.83 (0.13)	0.82	< 0.001
**Model 2:**									
Assessment (0 = *2017*, 1 = *2023*)	− 0.01 (0.05)	− 0.01	0.767	0.03 (0.05)	0.05	0.514	− 0.04 (0.05)	− 0.06	0.384
Reference: No NSSI									
Infrequent NSSI	− 0.01 (0.09)	0.00	0.873	− 0.06 (0.09)	− 0.05	0.500	0.13 (0.09)	0.10	0.141
Unstable repetitive NSSI	− 0.15 (0.12)	− 0.10	0.198	− 0.13 (0.11)	− 0.11	0.270	0.21 (0.11)	0.18	0.061
Stable repetitive NSSI	− 0.07 (0.15)	− 0.03	0.649	− 0.15 (0.15)	− 0.11	0.299	0.44 (0.14)	0.44	0.002
Psychological difficulties in adolescence	− 0.25 (0.04)	− 0.27	< 0.001	− 0.23 (0.04)	− 0.22	< 0.001	0.21 (0.04)	0.21	< 0.001
Gender (0 = *Boy/man*, 1 = *Girl/Woman*)	0.21 (0.08)	0.21	0.006	0.14 (0.08)	0.13	0.076	0.26 (0.07)	0.23	< 0.001

Note. NSSI frequency patterns were mutually exclusive dummy coded variables. Psychological difficulties were operationalized as the mean score over 2007–2008 for the Strength and Difficulties Questionnaire–self-report version. NSSI = Non-Suicidal Self-Injury

## Discussion

In this study, we examined changes in NSSI prevalence over approximately 15 years and whether different frequency patterns of NSSI engagement in adolescence (i.e., infrequent, unstable repetitive, and stable repetitive NSSI) were associated with NSSI and mental health in adulthood.

In line with previous research [9], the decrease in NSSI prevalence was less pronounced between young adulthood and adulthood proper (i.e., between 25 and 30 years old), than between adolescence and young adulthood (i.e., between 15 and 25 years old). Therefore, while NSSI becomes less prevalent with increasing age, the transition from adolescence to adulthood may–at the cohort level–involve a more pronounced shift in increased barriers to (e.g., social norms, improved self-concept) and reduced benefits from NSSI engagement (e.g., as a strategy for emotion regulation) than across the adulthood years (c.f [1]).

Regarding NSSI and mental health indicators in adulthood, we found that all frequency patterns of adolescent NSSI were associated with increased risk for any NSSI, and for elevated levels of depression, anxiety, and stress when other relevant factors were controlled for. This is in accordance with follow-up studies on younger samples (i.e., ages 18–25 [4, 5, 7]), and 35-year-olds [8]. Furthermore, repetitive adolescent NSSI (both unstable and stable) was predictive of lower life satisfaction and flourishing in a model that did not include other predictors. This is consistent with previous findings [11] of a negative association between adolescent NSSI and other well-being aspects (i.e., environmental mastery and self-acceptance) 10 years later. Additionally, we found a significant interaction effect indicating that individuals who reported a stable, repetitive NSSI pattern in adolescence and continued to self-injure retained a consistent level of NSSI engagement between young adulthood and adulthood proper. In contrast, individuals who reported infrequent or unstable repetitive NSSI during adolescence and continued to self-injure showed a reduction in their level of NSSI engagement over the same period. Furthermore, we found that stable repetitive NSSI was the only frequency pattern significantly associated with an increased risk for emotion dysregulation in adulthood, when controlling for other relevant factors. Thus, our findings suggest that adolescents with a stable repetitive NSSI pattern are more likely to experience ongoing difficulties in managing emotional reactions as well as persistent NSSI behavior into later stages of life.

To summarize, the findings of the present study suggest that adolescent NSSI is associated with psychological ill-being across the first decade of adulthood. The sensitivity of our multi-item NSSI frequency measure proved beneficial in identifying other adolescents who may experience mental health problems later in life, albeit to a lesser extent than those with more repetitive NSSI patterns.

### Strengths, limitations, and future directions

The current study, like many other longitudinal studies, encounters limitations due to attrition. While about 10% of our cohort did not respond to the survey in 2007–2008, this increased to 50% in 2017 and 65% in 2023. ALPAC (e.g [5]), and z-proso [12] have similarly recorded 50% attrition rates in adulthood. The differences between responders and non-responders in the observed variables, however, were small [20, 21]. We also applied statistical methods to balance for attrition, such as sample weighting and mixed modelling. Nevertheless, as registry data from ALPAC [6] suggested a higher rate of hospital admissions relating to self-harm (including suicidal behaviours) and mental health diagnoses among non-responders, there might still be bias in unobserved variables that could impact generalizability. Another limitation is the 10-year gap between measurements in adolescence and young adulthood. However, data collection in adolescence was subsequent of and/or coincided with the period when NSSI onset is most probable [13], which means that despite a lack of observations between 2009–2016, most cases are likely captured.

Despite these limitations, the current study offers valuable insights into the longitudinal outcomes of adolescent NSSI. Its importance is heightened by the fact that it stands as the only study spanning adolescence to adulthood that employs a multi-item measurement of NSSI, allowing for the differentiation between infrequent and repetitive NSSI. By including two measurement points in adolescence, we could also distinguish between NSSI engagement that is temporary or more stable. As a result, the current study sheds light on these frequency patterns’ association with mental health and well-being across the first decade of adulthood. There is only previously published follow-up study of adolescent NSSI where the sample is about 30 years of age or older (c.f [8]). However, studies that focus on risk at the group level are limited in their ability to draw conclusions about individual trajectories, such as whether individuals continue or cease to self-injure over time. To address this gap, future research should adopt a more person-oriented approach to track trajectories of NSSI engagement from adolescence into proper adulthood, enabling the study of how NSSI behaviors change on a case-by-case basis.

## Conclusions and clinical implications

The current study presents important findings on the prevalence of NSSI in adulthood proper and on longitudinal associations between adolescent NSSI and mental health in the first decade of adulthood. NSSI prevalence rates continuously declined from adolescence into adulthood proper, although the decline was less pronounced with increasing age. Engaging in NSSI during adolescence, particularly when NSSI was repetitive and stable across two measurement points, was associated with an increased risk for various mental health problems up to the age of 30. Nevertheless, weaker but significant associations were also observed with infrequent NSSI and unstable repetitive NSSI. Together, these findings underscore the importance of early intervention and support for adolescents engaging in any form of NSSI, regardless of frequency or stability, as well as the need for targeted interventions across various life stages. Additionally, proactive measures such as screening for various forms of self-injurious behaviors and recognizing that even a few affirmative answers can indicate a need for support are important. Moreover, our study underscores the need to develop and evaluate school-based interventions that promote help-seeking behaviors and foster the development of healthier emotion regulatory strategies, ultimately contributing to overall well-being over time.

## Electronic supplementary material

Below is the link to the electronic supplementary material.

Supplementary Material 1

## Data Availability

The data is available upon request from the corresponding author.

## Footnotes

1 This adjustment results in that subsample sizes and percentages are not equivalent to previous publications; however, percentages have not changed by more than a tithe, and it has not impacted the strength of any estimates.
