# Supplementary material for: Predicting adult mental health from non-suicidal self-injury in adolescence: a prospective study spanning 2007–2023
Source: Eur Child Adolesc Psychiatry. 2025 Jun 11;34(12):3791–800. doi: 10.1007/s00787-025-02785-8 (PMC12743085; doi:10.1007/s00787-025-02785-8)
Supplement: Supplementary file 1 — Supplementary Material 1 [file 787_2025_2785_MOESM1_ESM.docx]

**Table A1.**

*Descriptive statistics for the participants in 2017 and 2023.*

| Variable | | 2017 (*N* = 557) | 2023 (*N* = 386) |
| --- | --- | --- | --- |
| Age − *M* (SD) | | 25.3 (0.68) | 29.85 (0.75) |
| Gender – *n* (%) | |  |  |
|  | Woman | 327 (58.7%) | 243 (63%) |
|  | Man | 229 (41.1%) | 141 (36.5%) |
|  | Other/Do not want to disclose | 1 (0.2%) | 2 (0.5%) |
| Marital status – *n* (%) | |  |  |
|  | Single | 203 (36.4%) | 75 (19.4%) |
|  | Married/Cohabitant | 280 (50.3%) | 276 (71.5%) |
|  | In a relationship | 67 (12.0%) | 25 (6.5%) |
|  | Other | 7 (1.3%) | 10 (2.6%) |
| Child status – *n* (%) | |  |  |
|  | Yes | 58 (10.4%) | 140 (36.3%) |
|  | No | 498 (89.6%) | 246 (63.7%) |
| Educational level – *n* (%) | |  |  |
|  | Lower secondary education | 18 (3.2%) | 7 (1.8%) |
|  | Upper secondary school | 265 (47.7%) | 125 (32.4%) |
|  | Single university courses | 48 (8.6%) | 27 (7%) |
|  | University degree (<3 years) | 38 (6.8%) | 26 (6.7%) |
|  | University degree (≥3 years) | 172 (30.9%) | 189 (49%) |
|  | Other | 15 (2.7%) | 12 (3.1%) |
| Current employment status – *n* (%) | |  |  |
|  | Student | 145 (26.2%) | 28 (7.3%) |
|  | Full-/Part-time employment | 334 (60.4%) | 303 (78.5%) |
|  | Unemployed | 26 (4.7%) | 10 (2.6%) |
|  | On sick leave | 11 (2.0%) | 6 (1.6%) |
|  | On parental leave | 19 (3.4%) | 31 (8%) |
|  | Other | 18 (3.3%) | 8 (2.1%) |

*Note.* The project relies on self-reported gender such that participants could select if they identified as a *girl/woman* (2007−2023), *boy/man* (2007−2023), or *other/does not want to answer* (2017−2023). In the 5 instances of discriminant responses between 2007−2023, participant gender has been adjusted retrospectively to align with the most recent observation. This means that subsample sizes and percentages are not equivalent to previous publications; however, percentages have not changed by more than a tithe, and it has not impacted the strength of any estimates.

**Table A2.**

*Prevalence (%) and mean for NSSI in adulthood for the four adolescent NSSI frequency patterns.*

| Time of assessment | | Adolescent NSSI frequency pattern – *n*/*N* (%) or *M* (*SD*) | | | | | | | | | | | | | | |
| --- | --- | --- | --- | --- | --- | --- | --- | --- | --- | --- | --- | --- | --- | --- | --- | --- |
|  |  | No NSSI | | |  | Infrequent NSSI | | |  | Unstable repetitive NSSI | | |  | Repetitive NSSI | | |
|  |  | All | Women | Men |  | All | Women | Men |  | All | Women | Men |  | All | Women | Men |
| Any NSSI (≥1 instances) | |  |  |  |  |  |  |  |  |  |  |  |  |  |  |  |
|  | 2017 (T3) | 17/225 (7.6%) | 11/117 (9.4%) | 6/107 (5.6%) |  | 27/125 (21.6%) | 18/68 (26.5%) | 9/57 (15.8%) |  | 15/71 (21.1%) | 11/45 (24.4%) | 4/26 (15.4%) |  | 27/53 (50.9%) | 21/43 (48.8%) | 6/10 (60%) |
|  | 2023 (T4) | 12/149 (8.1%) | 5/86 (5.8%) | 6/62 (9.7%) |  | 19/96 (19.8%) | 12/57 (21.1%) | 7/39 (17.9%) |  | 8/55 (14.5%) | 7/35 (20%) | 1/19 (5.3%) |  | 13/32 (40.6%) | 11/28 (39.3%) | 2/4 (50%) |
| Repetitive NSSI (≥5 instances) | |  |  |  |  |  |  |  |  |  |  |  |  |  |  |  |
|  | 2017 (T3) | 11/225 (4.9%) | 6/117 (5.1%) | 5/107 (4.7%) |  | 11/125 (8.8%) | 6/68 (8.8%) | 5/57 (8.8%) |  | 6/71 (8.5%) | 4/45 (8.9%) | 2/26 (7.7%) |  | 19/53 (35.8%) | 14/43 (32.6%) | 5/10 (50%) |
|  | 2023 (T4) | 4/149 (2.7%) | 2/86 (2.3%) | 2/62 (3.2%) |  | 3/96 (3.1%) | 2/57 (3.5%) | 1/39 (2.6%) |  | 3/55 (5.5%) | 3/35 (8.6%) | 0/19 (0%) |  | 9/32 (28.1%) | 8/28 (28.6%) | 1/4 (25%) |
| NSSI, summed score | |  |  |  |  |  |  |  |  |  |  |  |  |  |  |  |
|  | 2017 (T3) | .72 (4.24) | .75 (3.74) | .70 (4.76) |  | 1.04 (3.06) | 1.37 (3.8) | .65 (1.79) |  | 1.20 (3.69) | 1.09 (2.72) | 1.38 (5.00) |  | 4.75 (7.60) | 4.56 (7.72) | 5.60 (7.40) |
|  | 2023 (T4) | .28 (1.26) | .22 (1.22) | .31 (1.29) |  | .54 (1.51) | .63 (1.72) | .41 (1.14) |  | .49 (1.45) | .71 (1.76) | .11 (0.46) |  | 4.88 (8.92) | 5.36 (9.42) | 1.50 (2.38) |

*Note*. Adulthood NSSI was assessed within the past 12 months. The frequency patterns were operationalized as follows: *no NSSI* (0 instances in the past 6 months in 2007 and 2008), *infrequent NSSI* (1−4 instances in the past 6 months in 2007 and/or 2008), *unstable repetitive NSSI* (≥5 instances in the past 6 months in 2007 or 2008), and *stable repetitive NSSI* (≥5 instances at in the past 6 months in 2007 and 2008). NSSI = Non-Suicidal Self-Injury.

**Table A3.**

*Exploratory interaction effects between time of assessment and NSSI frequency pattern in adolescence (2007−2008) in predicting NSSI in adulthood (2017-2023).*

|  |  | Zero-inflated component | | | |  | Poisson regression component | | |
| --- | --- | --- | --- | --- | --- | --- | --- | --- | --- |
| Predictor | | *α (SE)* | *OR* [95% *CI*] | *p* | *p*_bonferroni_ |  | *b* (*SE*) | *p* | *p*_bonferroni_ |
| Intercept | | 2.36 (.46) | 10.59 [4.28, 26.2] | <.001 | <.001 |  | 1.06 (1.11) | .341 | .999 |
| Assessment (0 = *2017*, 1 = *2023*) | | -.73 (.53) | .48 [.17, 1.35] | .166 | .999 |  | -1.91 (.24) | <.001 | <.001 |
| Reference: No NSSI | |  |  |  |  |  |  |  |  |
|  | Infrequent NSSI | -1.55 (.63) | .21 [.06, .73] | .014 | .136 |  | -.98 (.64) | .122 | .999 |
|  | Unstable repetitive NSSI | -1.15 (.46) | .32 [.13, .79] | .014 | .135 |  | -.42 (.57) | .464 | .999 |
|  | Stable repetitive NSSI | -2.24 (.57) | .11 [.04, .32] | <.001 | .001 |  | .26 (.54) | .624 | .999 |
| Gender (0 = *Boy/man*, 1 = *Girl/Woman*) | | -.23 (.14) | .79 [.60, 1.05] | .101 | .999 |  | .06 (.19) | .739 | .999 |
| Psychological difficulties | | -.33 (.35) | .72 [.36, 1.44] | .351 | .999 |  | -.01 (.44) | .990 | .999 |
| Reference: Assessment×No NSSI | |  |  |  |  |  |  |  |  |
|  | Assessment×Infrequent NSSI | .40 (.58) | 1.50 [.48, 4.70] | .488 | .999 |  | 1.13 (.30) | <.001 | .001 |
|  | Assessment×Unstable repetitive NSSI | .68 (.70) | 1.98 [.50, 7.77] | .327 | .999 |  | .91 (.30) | .002 | .021 |
|  | Assessment×Stable repetitive NSSI | 1.36 (.84) | 3.91 [.75, 2.38] | .106 | .999 |  | 2.51 (.47) | <.001 | <.001 |

*Note*. The zero-part predicts likelihood of *not* self-injuring. NSSI frequency patterns were mutually exclusive dummy coded variables. Psychological difficulties were operationalized as the mean score over 2007−2008 for the Strength and Difficulties Questionnaire−self-report version. NSSI = Non-suicidal self-injury

**Table A4.**

*Exploratory interaction effects between time of assessment and NSSI frequency pattern in adolescence (2007−2008) in predicting health outcomes and emotion regulation in adulthood (2017−2023).*

| Outcome/Predictor | | | *β* (*SE*) | *β*_robust_ | *p* | *p*_bonferroni_ |
| --- | --- | --- | --- | --- | --- | --- |
| **Depression** | | |  |  |  |  |
|  | Intercept | | -.22 (.07) | -.34 | .003 | .030 |
|  | Assessment (0 = *2017*, 1 = *2023*) | | -.05 (.09) | -.01 | .569 | .999 |
|  | Reference: No NSSI | |  |  |  |  |
|  |  | Infrequent NSSI | .29 (.10) | .22 | .006 | .060 |
|  |  | Unstable repetitive NSSI | .29 (.13) | .26 | .025 | .250 |
|  |  | Stable repetitive NSSI | .19 (.16) | .18 | .218 | .999 |
|  | Psychological difficulties in adolescence | | .18 (.04) | .15 | <.001 | <.001 |
|  | Gender (0 = *Boy/man*, 1 = *Girl/Woman*) | | .11 (.07) | .11 | .141 | .999 |
|  | Reference: Assessment×No NSSI | |  |  |  |  |
|  |  | Assessment×Infrequent NSSI | -.11 (.14) | -.09 | .414 | .999 |
|  |  | Assessment×Unstable repetitive NSSI | .14 (.17) | .07 | .403 | .999 |
|  |  | Assessment×Stable repetitive NSSI | .26 (.20) | .10 | .195 | .999 |
| **Anxiety** | | |  |  |  |  |
|  | Intercept | | -.27 (.08) | -.34 | <.001 | <.001 |
|  | Assessment (0 = *2017*, 1 = *2023*) | | .03 (.08) | -.02 | .705 | .999 |
|  | Reference: No NSSI | |  |  |  |  |
|  |  | Infrequent NSSI | .26 (.10) | .16 | .014 | .140 |
|  |  | Unstable repetitive NSSI | .39 (.13) | .32 | .003 | .030 |
|  |  | Stable repetitive NSSI | .34 (.16) | .36 | .031 | .310 |
|  | Psychological difficulties in adolescence | | .17 (.04) | .13 | <.001 | <.001 |
|  | Gender (0 = *Boy/man*, 1 = *Girl/Woman*) | | .15 (.07) | .10 | .041 | .410 |
|  | Reference: Assessment×No NSSI | |  |  |  |  |
|  |  | Assessment×Infrequent NSSI | -.13 (.14) | .02 | .365 | .999 |
|  |  | Assessment×Unstable repetitive NSSI | -.21 (.17) | -.14 | .201 | .999 |
|  |  | Assessment×Stable repetitive NSSI | .16 (.20) | .08 | .412 | .999 |
| **Stress** | | |  |  |  |  |
|  | Intercept | | -.33 (.08) | -.38 | <.001 | <.001 |
|  | Assessment (0 = *2017*, 1 = *2023*) | | -.03 (.08) | -.02 | .750 | .999 |
|  | Reference: No NSSI | |  |  |  |  |
|  |  | Infrequent NSSI | .19 (.10) | .14 | .072 | .720 |
|  |  | Unstable repetitive NSSI | .30 (.13) | .29 | .019 | .190 |
|  |  | Stable repetitive NSSI | .37 (.16) | .35 | .018 | .180 |
|  | Psychological difficulties in adolescence | | .19 (.04) | .20 | <.001 | <.001 |
|  | Gender (0 = *Boy/man*, 1 = *Girl/Woman*) | | .34 (.07) | .32 | <.001 | <.001 |
|  | Reference: Assessment×No NSSI | |  |  |  |  |
|  |  | Assessment×Infrequent NSSI | .00 (.14) | .01 | .992 | .999 |
|  |  | Assessment×Unstable repetitive NSSI | .03 (.16) | .03 | .844 | .999 |
|  |  | Assessment×Stable repetitive NSSI | -.26 (.19) | -.35 | .176 | .999 |
| **Life satisfaction** | | |  |  |  |  |
|  | Intercept | | -.09 (.08) | -.06 | .233 | .999 |
|  | Assessment (0 = *2017*, 1 = *2023*) | | .04 (.07) | .02 | .616 | .999 |
|  | Reference: No NSSI | |  |  |  |  |
|  |  | Infrequent NSSI | -.01 (.10) | .00 | .936 | .999 |
|  |  | Unstable repetitive NSSI | -.10 (.13) | -.05 | .440 | .999 |
|  |  | Stable repetitive NSSI | .03 (.16) | .04 | .848 | .999 |
|  | Psychological difficulties in adolescence | | -.26 (.04) | -.27 | <.001 | <.001 |
|  | Gender (0 = *Boy/man*, 1 = *Girl/Woman*) | | .21 (.08) | .21 | .006 | .060 |
|  | Reference: Assessment×No NSSI | |  |  |  |  |
|  |  | Assessment×Infrequent NSSI | -.02 (.12) | .01 | .880 | .999 |
|  |  | Assessment×Unstable repetitive NSSI | -.12 (.15) | -.11 | .423 | .999 |
|  |  | Assessment×Stable repetitive NSSI | -.27 (.17) | -.18 | .116 | .999 |

**Table A4 continued.**

| Outcome/Predictor | | | *β* (*SE*) | *β*_robust_ | *p* | *p*_bonferroni_ |
| --- | --- | --- | --- | --- | --- | --- |
| **Flourishing** | | |  |  |  |  |
|  | Intercept | | -.04 (.08) | .02 | .626 | .999 |
|  | Assessment (0 = *2017*, 1 = *2023*) | | .07 (.07) | .08 | .311 | .999 |
|  | Reference: No NSSI | |  |  |  |  |
|  |  | Infrequent NSSI | -.10 (.10) | -.09 | .321 | .999 |
|  |  | Unstable repetitive NSSI | -.01 (.13) | .00 | .952 | .999 |
|  |  | Stable repetitive NSSI | -.07 (.16) | -.05 | .640 | .999 |
|  | Psychological difficulties in adolescence | | -.23 (.04) | -.23 | <.001 | <.001 |
|  | Gender (0 = *Boy/man*, 1 = *Girl/Woman*) | | .14 (.08) | .13 | .079 | .790 |
|  | Reference: Assessment×No NSSI | |  |  |  |  |
|  |  | Assessment×Infrequent NSSI | .10 (.12) | .09 | .405 | .999 |
|  |  | Assessment×Unstable repetitive NSSI | -.28 (.14) | -.26 | .045 | .450 |
|  |  | Assessment×Stable repetitive NSSI | -.21 (.17) | -.15 | .215 | .999 |
| **Emotion dysregulation** | | |  |  |  |  |
|  | Intercept | | -.29 (.07) | -.33 | <.001 | <.001 |
|  | Assessment (0 = *2017*, 1 = *2023*) | | -.01 (.07) | -.01 | .878 | .999 |
|  | Reference: No NSSI | |  |  |  |  |
|  |  | Infrequent NSSI | .15 (.10) | .11 | .141 | .999 |
|  |  | Unstable repetitive NSSI | .22 (.13) | .21 | .077 | .770 |
|  |  | Stable repetitive NSSI | .48 (.15) | .52 | .002 | .020 |
|  | Psychological difficulties in adolescence | | .21 (.04) | .21 | <.001 | <.001 |
|  | Gender (0 = *Boy/man*, 1 = *Girl/Woman*) | | .26 (.07) | .23 | <.001 | <.001 |
|  | Reference: Assessment×No NSSI | |  |  |  |  |
|  |  | Assessment×Infrequent NSSI | -.04 (.12) | -.04 | .710 | .999 |
|  |  | Assessment×Unstable repetitive NSSI | -.04 (.14) | -.07 | .801 | .999 |
|  |  | Assessment×Stable repetitive NSSI | -.14 (.17) | -.22 | .426 | .999 |

*Note*. NSSI frequency patterns were mutually exclusive dummy coded variables. Psychological difficulties were operationalized as the mean score over 2007−2008 for the Strength and Difficulties Questionnaire−self-report version. NSSI = Non-Suicidal Self-Injury.
